# Supplementary material for: nlive: an R package to facilitate the application of the sigmoidal and random changepoint mixed models
Source: BMC Med Res Methodol. 2023 Nov 3;23:257. doi: 10.1186/s12874-023-02075-4 (PMC10623729; doi:10.1186/s12874-023-02075-4)
Supplement: Supplementary file 1 — Additional file 1. Supplementary materials. [file 12874_2023_2075_MOESM1_ESM.pdf]

# SUPPLEMENTARY MATERIALS

**APPENDIX 1**. Call of the original monolithic `nlive()` function implemented in the package to fit the SMM, PMM-abrupt, PMM-smooth models.

```
nlive(model, dataset, ID, outcome, time, predictor.all=NULL, predictor.par1=NULL,
predictor.par2=NULL, predictor.par3=NULL, predictor.par4=NULL)
```

The first five arguments are mandatory, while all the others have default values. Below is a brief description of the arguments:

- `model`: indicator of the model to fit (1=SMM, 2=abrupt PMM, 3=smooth PMM).
- `dataset`: data frame containing the variables named in `ID`, `outcome`, `time`, `predictor.all`, and `predictor.par1` to `predictor.par4`.
- `ID`: name of the variable representing the grouping structure specified with " (e.g., "ID" representing the unique identifier of participants).
- `outcome`: name of the time-varying variable representing the longitudinal outcome specified with " (e.g., "outcome")
- `time`: name of the variable representing the timescale specified with " (e.g., "time"). Can be negative or positive. Note that model 1, SMM, will always report a positive value under the midpoint parameter (e.g. if the time in the data goes from 0 down to -10 and the time of the midpoint is -2, the model will report a midpoint of 2).
- `predictor.all`: optional vector indicating the name of the variable(s) that the four main parameters of the model will be adjusted to (e.g. `predictor.all = c("X1","X2")`). Default to NULL.
- `predictor.par1`: optional vector indicating the name of the variable(s) that the first main parameter of the model will be adjusted to (e.g. `predictor.all = c("X1","X2")`). For model 1, the first parameter = last level. For models 2 and 3, first parameter = intercept. Default to NULL.
- `predictor.par2`: optional vector indicating the name of the variable(s) that the second main parameter of the model will be adjusted to (e.g. `predictor.all = c("X1","X2")`). For model 1, the second parameter = initial level. For models 2 and 3, second parameter = slope1 (slope before the change-point). Default to NULL.
- `predictor.par3`: optional vector indicating the name of the variable(s) that the third main parameter of the model will be adjusted to (e.g. `predictor.all = c("X1","X2")`). For model 1, the third parameter = midpoint. For models 2 and 3, third parameter = slope2 (slope between intercept and changepoint). Default to NULL.
- `predictor.par4`: optional vector indicating the name of the variable(s) that the fourth main parameter of the model will be adjusted to (e.g. `predictor.all = c("X1","X2")`). For model 1, the fourth parameter is the Hill slope. For models 2 and 3, the fourth parameter is the changepoint. Default to NULL.

**APPENDIX 2**. R code to replicate spaghetti plots and boxplots.

```
- Spaghetti plot -
R> first = as.data.frame(dataCog %>% group_by(ID) %>% filter(row_number(ID) == 1))
R> tempo1 = sample_n(first, 70)
R> tempo2 = sqldf('SELECT * FROM dataCog WHERE ID IN (SELECT ID FROM tempo1)')
R> ggplot(tempo2, aes(time, cognition, group = ID, colour = factor(ID))) +
geom_line(size = 0.8) +
scale_x_continuous(limits = c(-25, 0)) +
guides(colour = "none") +
labs(title = "Spaghetti plot, random selection of 70 individuals")
```

```
- Boxplots -
R> dataCog$time_box = round(dataCog$time, 0)
R> ggplot(dataCog, aes(x = time_box, y = cognition)) +
geom_boxplot(aes(fill = as.factor(time_box))) +
scale_y_continuous(limits = c(-25, 0)) +
labs(title = "Boxplots over time, whole sample") +
theme(legend.position = "none")
```

**APPENDIX 3**. Main output of `nlive.smm()`, corresponding to the general output from `saemix`.

```
Nonlinear mixed-effects model fit by the SAEM algorithm
-----------------------------------
----            Data             ----
-----------------------------------
Object of class SaemixData
    longitudinal data for use with the SAEM algorithm
Dataset C:/Document/dataset.txt
    Structured data: cognition ~ time | ID
    Predictor: time ()
    covariates: ageDeath90 (-)
Dataset characteristics:
    number of subjects:     1200
    number of observations: 11867
    average/min/max nb obs: 9.89  /  2  /  24
First 10 lines of data:
     ID   time cognition ageDeath90 mdv cens occ ytype
1  1000 -10.00      0.45          1   0    0   1     1
2  1000  -9.08      0.27          1   0    0   1     1
3  1000  -8.04      0.19          1   0    0   1     1
4  1000  -6.82      0.15          1   0    0   1     1
5  1000  -5.99      0.05          1   0    0   1     1
6  1000  -4.98      0.15          1   0    0   1     1
7  1000  -3.82     -0.21          1   0    0   1     1
8  1000  -2.93      0.68          1   0    0   1     1
9  1000  -2.16      0.32          1   0    0   1     1
10 1000  -1.10     -0.49          1   0    0   1     1
-----------------------------------
----            Model            ----
-----------------------------------
Nonlinear mixed-effects model
  Model function
  Model type:  structural
function(psi, ID, xidep){
    t           = xidep[, 1]
    last.level  = psi[ID, 1]
    first.level = psi[ID, 2]
    midpoint    = psi[ID, 3]
    hill.slope  = psi[ID, 4]
    t2 = abs(t)
    Y_pred = SSlogis5(t2, last.level, first.level, midpoint, hill.slope, theta = 1)
    return(Y_pred)
  }
  Nb of parameters: 4
      parameter names:  last.level first.level midpoint hill.slope
      distribution:
     Parameter   Distribution Estimated
[1,] last.level  normal       Estimated
[2,] first.level normal       Estimated
```

```
[3,]  midpoint    normal      Estimated
[4,]  hill.slope  normal      Estimated
  Variance-covariance matrix:
            last.level first.level midpoint hill.slope
last.level           1           1        0          0
first.level          1           1        0          0
midpoint             0           0        0          0
hill.slope           0           0        0          0
  Error model: constant , initial values: a.1=1
  Covariate model:
           [,1] [,2] [,3] [,4]
ageDeath90    1    1    1    1
    Initial values
             last.level first.level midpoint hill.slope
Pop.CondInit  -1.015734   0.1384007        2        0.5
Cov.CondInit   0.000000   0.0000000        0        0.0
----------------------------------------------
----      Key algorithm options      ----
----------------------------------------------
    Estimation of standard errors and linearised log-likelihood
    Estimation of log-likelihood by importance sampling
    Number of iterations:  K1=300, K2=100
    Number of chains:  1
    Seed:  123
    Number of MCMC iterations for IS:  5000
    Simulations:
        nb of simulated datasets used for npde:  1000
        nb of simulated datasets used for VPC:  100
    Input/output
        save the results to a file:  FALSE
        save the graphs to files:  FALSE
------------------------------------------------------------
----                    Results                    ----
------------------------------------------------------------
-----------------  Fixed effects  -----------------
------------------------------------------------------------
      Parameter                     Estimate SE     CV(%)   p-value
 [1,] last.level                    -1.07501 0.0346    3.22 -
 [2,] beta_ageDeath90(last.level)   -0.06119 0.0043    7.10 0.0e+00
 [3,] first.level                    0.22334 0.0143    6.39 -
 [4,] beta_ageDeath90(first.level)  -0.04176 0.0018    4.23 0.0e+00
 [5,] midpoint                       2.56061 0.0330    1.29 -
 [6,] beta_ageDeath90(midpoint)      0.03406 0.0042   12.45 4.4e-16
 [7,] hill.slope                     1.86198 0.0417    2.24 -
 [8,] beta_ageDeath90(hill.slope)    0.00016 0.0054 3449.73 4.9e-01
 [9,] a.1                            0.27899 0.0020    0.72 -
------------------------------------------------------------
-----------  Variance of random effects  -----------
------------------------------------------------------------
            Parameter                Estimate SE    CV(%)
last.level  omega2.last.level        1.262    0.055  4.3
```

```
first.level omega2.first.level          0.144     0.007   4.9
covar       cov.last.level.first.level 0.065     0.014  21.7
-----------------------------------------------------------
-------  Correlation matrix of random effects  -------
-----------------------------------------------------------
                    omega2.last.level omega2.first.level
omega2.last.level   1.00              0.15
omega2.first.level  0.15              1.00
-----------------------------------------------------------
----------------  Statistical criteria  --------------
-----------------------------------------------------------
Likelihood computed by linearisation
      -2LL= 9723.199
      AIC = 9747.199
      BIC = 9808.279

Likelihood computed by importance sampling
      -2LL= 9727.557
      AIC = 9751.557
      BIC = 9812.638
-----------------------------------------------------------
                        Parameter Estimate    SE p-value
1                      last.level   -1.088 0.035 P<.0001
2   beta_ageDeath90(last.level)    -0.061 0.004 P<.0001
3                     first.level    0.24 0.015 P<.0001
4  beta_ageDeath90(first.level)    -0.044 0.002 P<.0001
5                        midpoint   -2.567 0.034 P<.0001
6     beta_ageDeath90(midpoint)     0.031 0.004 P<.0001
7                      hill.slope    1.789  0.04 P<.0001
8   beta_ageDeath90(hill.slope)     0.007 0.005   0.081
9                           error    0.279 0.002 P<.0001
-----------------------------------------------------------
 The program took 346.51 seconds
```
